# Phenotypic Heterogeneity of Post-lingual and/or Milder Hearing Loss for the Patients With the GJB2 c.235delC Homozygous Mutation

**DOI:** 10.3389/fcell.2021.647240

**Published:** 2021-02-26

**Authors:** Hongyang Wang, Yun Gao, Jing Guan, Lan Lan, Ju Yang, Wenping Xiong, Cui Zhao, Linyi Xie, Lan Yu, Dayong Wang, Qiuju Wang

**Affiliations:** ^1^College of Otolaryngology, Head and Neck Surgery, Chinese People’s Liberation Army (PLA) Institute of Otolaryngology, Chinese People’s Liberation Army (PLA) General Hospital, Beijing, China; ^2^National Clinical Research Center for Otolaryngologic Diseases, Beijing, China; ^3^Key Lab of Hearing Impairment Science of Ministry of Education, Beijing, China; ^4^Key Lab of Hearing Impairment Prevention and Treatment of Beijing, Beijing, China

**Keywords:** GJB2, Connexin30, modifier, heterogeneity, hearing loss

## Abstract

**Objective:**

To report the phenotypic heterogeneity of *GJB2* c.235delC homozygotes associated with post-lingual and/or milder hearing loss, and explore the possible mechanism of these unconditional phenotypes.

**Methods:**

Mutation screening of *GJB2* was performed on all ascertained members from Family 1006983 and three sporadic patients by polymerase chain reaction (PCR) amplification and Sanger sequencing. Next generation sequencing (NGS) was successively performed on some of the affected members and normal controls from Family 1006983 to explore additional possible genetic codes. Reverse transcriptase–quantitative PCR was conducted to test the expression of Connexin30.

**Results:**

We identified a Chinese autosomal recessive hearing loss family with the *GJB2* c.235delC homozygous mutation, affected members from which had post-lingual moderate to profound hearing impairment, and three sporadic patients with post-lingual moderate hearing impairment, instead of congenital profound hearing loss. NGS showed no other particular variants. Overexpression of Connexin30 in some of these cases was verified.

**Conclusion:**

Post-lingual and/or moderate hearing impairment phenotypes of *GJB2* c.235delC homozygotes are not the most common phenotype, revealing the heterogeneity of *GJB2* pathogenic mutations. To determine the possible mechanism that rescues part of the hearing or postpones onset age of these cases, more cases are required to confirm both Connexin30 overexpression and the existence of modifier genes.

## Introduction

Hearing loss is one of the major disabilities world-widely, which is often induced by loss of sensory hair cells (HCs) in the inner ear cochlea. HCs mainly function in transducing sound waves into the electric signals (Wang et al., 2017; Liu et al., 2019; Qi et al., 2019; He et al., 2020). Hearing loss could be caused by genetic factors, aging, chronic cochlear infections, infectious diseases, ototoxic drugs, and noise exposure (He et al., 2017; Cheng et al., 2019; Zhou et al., 2020); and genetic factors account for more than 60% of the hearing loss. *GJB2*, which encodes the gap junction protein Connexin 26, is the most common cause of genetic non-syndromic hearing loss (NSHL). Among the approximately 200 hearing loss associated *GJB2* mutations (Stenson et al., 2017), the c.235delC mutation, a founder mutation (Yan et al., 2003; Shinagawa et al., 2020), is the most frequently known mutation in some East Asian groups, with a carrier frequency of approximately 1% (Yan et al., 2003). The mutation has a significant ethnic specificity, with increasing risk of NSHL in the East Asian and Southeast Asian populations (Dai et al., 2015), but no susceptibility in Oceania and European populations (Yao et al., 2012). Most patients with the *GJB2* c.235delC homozygous mutation show pre-lingual severe to profound hearing impairment (Zhao et al., 2011). However, even cases with the *GJB2* c.235delC homozygous mutation may be undetected by newborn hearing screening, suggesting that *GJB2* c.235delC homozygous mutation-related hearing loss may not always be congenital at onset (Minami et al., 2013; Dai et al., 2019; Wang et al., 2019). Clinically, except for late-onset hearing impairment, some *GJB2* c.235delC homozygous patients show post-lingual and/or mild to moderate hearing impairment instead of profound hearing loss with a highly heterogeneous phenotype.

In addition to *GJB2, GJB6* is another gene located in DFNB1. *GJB6* encodes the gap junction protein Connexin 30, which is co-assemble with Connexin 26 to form hybrid gap junctions (Ahmad et al., 2003). Connexin26 and Connexin 30 are predominant isoforms in the cochlea (Zhao et al., 2006). Deletions in *GJB6* are also common genetic factors for NSHL in some populations, with ethnic specificity (del Castillo et al., 2002; Marlin et al., 2005; Falah et al., 2020). Cases with digenic heterozygous mutations in *GJB2* and *GJB6* are relatively rare in the NSHL population, with various phenotypes, such as prelingual or post-lingual, ranging from mild or moderate to severe or profound hearing loss (Bolz et al., 2004; Cama et al., 2009; Chan et al., 2010; del Castillo and del Castillo, 2011; Mei et al., 2017). In the *GJB6* homozygous knockout mouse model, overexpression of Connexin26 restored hearing sensitivity and prevented hair cell death, suggesting that upregulation of Connexin might be a therapeutic strategy for patients with *GJB6* mutations (Ahmad et al., 2007). However, there is no animal experiment to verify that the overexpression of Connexin30 can restore the hearing loss caused by *GJB2* mutations, since *Gjb2* knockout mice are embryonically lethal (Gabriel et al., 1998).

Modifier genes are a perspective for the study of hearing loss to interpret phenotypic variation. Linkage analysis (families) and association studies (unrelated patients) are two strategies can be adopted to identify modifier genes for human disorders (Bykhovskaya et al., 2004; Dunckley et al., 2007; Tao et al., 2019). However, association studies are not always effective, and a previous whole genome association (WGA) study on *GJB2* c.35delG homozygous patients showed that phenotypic heterogeneity could not be explained by the effect of a single major modifier gene (Azaiez et al., 2004; Hilgert et al., 2009a).

In this study, we focused on some unconditional cases involving *GJB2* c.235delC homozygotes, whose phenotypes were late-onset and/or moderate hearing loss. First, we found a Chinese family (Family 1006983) with the *GJB2* c.235delC mutation, which was segregated with all of the recruited members. Unlike other patients with the *GJB2* c.235delC homozygous mutation, whose phenotype were pre-lingual severe to profound hearing loss, patients in this family showed post-lingual moderate to profound sensorineural hearing loss. In addition, we further identified three sporadic cases with *GJB2* c.235delC homozygotes, the phenotypes of which were post-lingual moderate hearing impairment. To decipher the mechanism of partial or late-onset hearing loss in *GJB2* c.235delC homozygotes cases, we performed next generation sequencing (NGS), including targeted genes capture and genome sequencing on some of the affected members and controls from 1006983, but no pathogenic variations or modifier genes were identified. Since Connexin26 and Connexin 30 are co-assembled to form hybrid gap junctions and a previous mouse experiment suggested that up-regulation of Connexin or the slowing of its degradation might be a therapeutic strategy to prevent and treat deafness caused by Connexin 30 mutations (Ahmad et al., 2007), we hypothesised that for some of the patients with a more minor phenotype than other patients with the *GJB2* c.235delC mutation, it is the overexpression of Connexin30 that plays a compensatory role. Thus, reverse transcriptase-quantitative polymerase chain reaction (RT-qPCR) was also conducted on some of these patients to test the expression of Connexin30, which is encoded by *GJB6* (Supplementary Figure 1).

## Materials and Methods

### Ethics Statement

The study was approved by the Committee of Medical Ethics of the Chinese PLA General Hospital. Written informed consents were obtained from all the participants in the family.

### Family Recruitment and Clinical Evaluations

**FIGURE 1:**
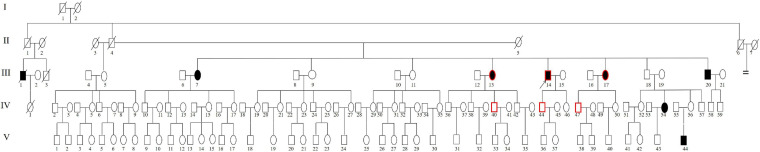
Pedigree of Family 1006983. Filled symbols for males (squares) and females (circles) represent affected individuals, and empty, unaffected individuals. An arrow denotes the proband. The symbols with a red border represent the cases for whom genome sequencing was performed.

**FIGURE 2:**
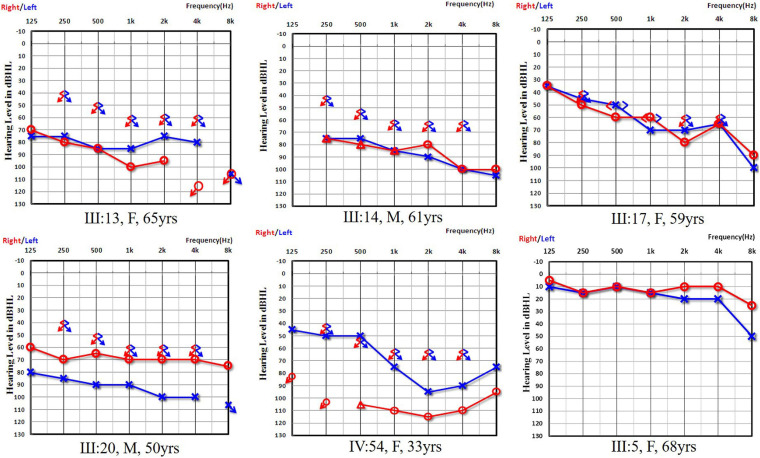
Audiograms of both ears from subjects in Family 1006983. Symbols “o” and “x” denote air conduction pure-tone thresholds at different frequencies in the right and left ear, respectively. dB HL, decibels hearing level; Hz, Hertz. III:13, III:14, III:17, III:20, and IV:54 are the affected cases from Family 1006983 with the *GJB2* c.235delC homozygous mutation. III:5 is a normal control from the family with a *GJB2* c.235delC heterozygous mutation.

**FIGURE 3:**
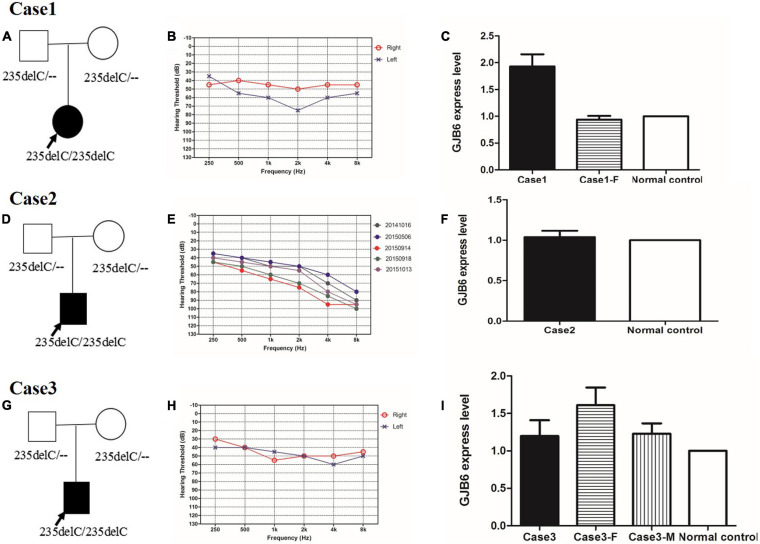
**(A,D,G)** Pedigree of case 1, case 2, and case 3, respectively. **(B,E,H)** Audiograms of three sporadic cases. **(B)** Audiograms of both ears from case 1. **(E)** Five audiograms of the left ear from case 2 in the last 2 years. Different color audiograms represent audiograms done at different times. **(H)** Audiograms of both ears from case 3. **(B,E,H)** Symbols “o” and “x” denote air conduction pure-tone thresholds at different frequencies in the right and left ear, respectively. dB, decibels; Hz, Hertz. **(C,F,I)** Connexin 30 expression in case 1, case 2, and case 3, respectively. Case 1-F is the father of case 1. Case3-F is the father of case 3, and Case 3-M is the mother of Case 3. Normal control is an independent subject with normal hearing from our institute.

A five-generation family (Family 1006983) with 133 members presenting with autosomal recessive non-syndromic sensorineural hearing loss (ARNSHL) was ascertained from the Institute of Otolaryngology, Chinese PLA General Hospital (Figures 1, 2). Three independent sporadic patients with homozygous *GJB2* c.235delC were also recruited in our study, who had late-onset moderate hearing impairment (Figure 3). Personal or family medical evidence of hearing loss, tinnitus, vestibular symptoms, and other clinical abnormalities of the participants were identified by a team of experienced physicians and audiologists. Audiometric evaluations included pure tone audiometry (PTA), which was calculated as the average hearing threshold at 0.5, 1, 2, and 4 kHz for the bilateral ears of the ascertained subjects. The severity of hearing impairment was defined as mild (26–40 dB HL), moderate (41–70 dB HL), severe (71–95 dB HL) and profound (>95 dB HL). The speech recognition score was tested and calculated for some patients. Caloric testing was performed on some patients and the normal controls to obtain data on semicircular canal function. Some patients who had tinnitus were estimated by tinnitus assessment. High resolution computed tomography (HRCT) of the temporal bone was conducted for some of the patients.

A cohort of two hundred and ninety-five hearing loss cases who had the homozygous *GJB2* c.235delC mutation in our institute were chosen as the controls to compare phenotypes (Zhao et al., 2011).

### Sanger Sequencing

Genomic DNA was extracted from the whole blood samples using a Blood DNA kit according to the standard protocol (TIANGEN BIOTECH, Beijing, China). PCR and Sanger sequencing were performed on all available members from Family 1006983 and the three sporadic patients and their parents to determine whether the potential mutations in causative genes co-segregated with the disease phenotype in these families. Direct PCR products were sequenced using Bigdye Terminator v3.1 Cycle Sequencing Kits (Applied Biosystems, Foster City, CA) and analysed using an ABI 3700XL Genetic Analyser (primers were *GJB2*-Exon2-F: 5′-TTGGTGTTTGCTCAGGAAGA-3′ and *GJB2*-Exon2-R: 5′-GGCCTACAGGGGTTTCAAAT-3′).

### Next Generation Sequencing

To examine whether other genetic defects or modifier genes were involved, targeted multi-genes capture and NGS were performed on case III:20, who had moderate hearing impairment in the better ear at the age of 50 years when tested. Targeted genes include 307 known and candidate hearing loss associated genes (Wang et al., 2015). And then genome sequencing was further performed on cases III:13, III:14, III:17 and controls IV;40, IV;44, IV:47. Sequencing was carried out on Illumina HiSeq2500 to generate paired end reads. Biologic information analysis was carried out according to a previous process (Wang et al., 2014), and specific analysis strategies to find the modifier genes were conducted on the genome sequencing data.

### RT-qPCR of GJB6

Total RNA was extracted from the white cells of the whole blood samples using the cell RAN kit according to the standard protocol (TRIzol Reagent, Life Technologies). Synthesis of cDNA was carried out with a Revert Aid First Strand cDNA Synthesis Kit (Thermo Fisher Scientific). Primers were designed as follows by Primer5: GAPDH-H-F: GGAGCGAGATCCCTCCAAAAT; GAPDH-H-R: GGAGCGAGATCCCTCCAAAAT; GJB6-H-F: CAAGAGGACTTCGTCTGCAAC; GJB6-H-R: GTGGTTTCGTGCCTGTAGTAG. RT-qPCR was conducted according to the protocol of the “Power SYBR Green PCR Master Mix and RT-PCR” by Applied Biosystems. The quantitative analysis conditions were shown in Supplementary Figure 2. Expression stability analysis included geNorm analysis, NormFinder analysis and BestKeeper analysis. Statistical analysis was performed using Graphpad Prism version 5.01. All of the quantitative results were duplicated at least twice.

## Results

### Clinical Description

In Family 1006983, a total of 22 family members, composed of five clinically affected and 17 unaffected individuals were ascertained in this study. In this family, most of the affected members showed post-lingual, approximately symmetrical, and bilateral non-syndromic sensorineural hearing loss. The average onset age for all the affected cases was 6.2 years old. The hearing loss was presented at all frequencies (Table 1 and Figure 2). For the other three independent sporadic patients in this study, case 1 was a 5 years old girl who had post-lingual, bilateral symmetrical moderate sensorineural hearing impairment, with an onset age of 5 years old (Figures 3A,B); case 2 was a 37-year-old man suffering from sudden hearing loss of left ear accompanied by dizziness and tinnitus when he visited our hospital in 2015, whose hearing threshold had a fluctuation in the last 2 years, and whose onset age was not clear. His hearing threshold was first tested on 16th October, 2014, and the PTAs of the left and right ear were 52.5 and 56.25 dB HL, respectively (Figures 3D,E); Case 3 was a 6 year old boy with post-lingual, bilateral symmetrical moderate sensorineural hearing impairment (Figures 3G,H). All of these three cases had no family history of hearing loss, no symptoms in other organ systems and no other exposure to risk factors. All of the affected cases had associated tinnitus, but no vestibular symptoms or signs were reported except for case 2. HRCT of the temporal bone in the proband of Family 1006983 (III:14) and three sporadic cases showed normal inner ears structure. No exposure history that may account for the hearing impairment existed in any of the affected members.

**TABLE 1:** Summary of clinical data for hearing impaired members in Family 1006983 and three sporadic cases.

**Subject**	**Gender^a^**	**Age of test (year)**	**Age of onset (year)**	**PTA (dB HL)^b^**	**Hearing impairment^c^**	**Audiogram**	**Tinnitus**	**Vertigo**
III:13 (−3)	F	65	5	L: 81.25	Severe	Flat	+	−
				R: 98.75	Profound	Flat		
III:14 (−4)	M	61	6	L: 87.5	Severe	Flat	+	−
				R: 86.25	Severe	Flat		
III:17 (−7)	F	59	9	L: 63.75	Moderate	Flat	+	−
				R: 66.25	Moderate	Flat		
III:20 (−5)	M	50	7	L: 96.25	Profound	Flat	+	−
				R: 68.75	Moderate	Flat		
IV:54 (−67)	F	33	5	L: 77.5	Severe	Flat	+	−
				R: 110	Profound	Flat		
Case1	F	5	5	L: 45	Moderate	Flat	+	−
				R: 62.5	Moderate	Flat		
Case2	M	37	Not clear	L: 57.5	Moderate	Flat	+	+
				R: 55	Moderate	Flat		
Case3	M	6	6	L: 48.75	Moderate	Flat	+	−
				R: 48.75	Moderate	Flat		

*^*a*^M, male; F, female. ^*b*^PTA, pure-tone air-conduction averages (0.5, 1, 2, and 4 kHz). ^*c*^Diagnosed at the time of test (case3 showed the last test on 13th October, 2015). +, positive finding;* −, *negative finding.*

Among the 295 cases with the homozygous *GJB2* c.235delC mutation, four patients had late-onset hearing loss, an incidence of 1.36% (4/295) (Zhao et al., 2011). The onset ages of these four cases were 4, 4, 5, and 6 years old, with an average onset age of 4.75 years old. Among these four c.235delC homozygous cases, three children had severe hearing loss, and one had profound hearing impairment. There were also eight patients who had pre-lingual moderate hearing loss, an incidence of 2.71% (8/295).

### Mutation Detection and Analysis

The c.235delC is a known pathogenic ARNSHL related mutation. Sanger sequencing confirmed the co-segregation of c.235delC with the disease phenotype in Family 1006983, the three sporadic patients and their parents (Figures 3A,D,G). All of the cases with hearing impairment had homozygous c.235delC mutation, and all of the normal controls recruited in this study had either no mutation or heterozygous c.235delC. The genotype frequency in dbSNP137, HapMap, 1,000 Genomes, and local dataset was less than 0.001. This mutation occurred at highly conserved amino acids, and was predicted to be deleterious by the PolyPhen 2, SIFT, and Mutationtaster programs.

A total of 307 hearing loss related genes were captured, and the targeted high-throughput sequencing data were analysed according to the standard process (Wang et al., 2014) and no pathogenic or likely pathogenic variations were identified according to the standards and guidelines for the interpretation of sequence variants (Richards et al., 2015).

To further analyse the modified genes that may exist in the 1006983 family, six samples from the family were selected for genome sequencing analysis. The total amount of data of the whole genome was shown in Supplementary Table 1. After screening, a total of 7,132,601 digits of variation were obtained. A total of 6.6% of them were novel variants. Sample sequencing depth and coverage are shown in Supplementary Table 2. Variation covers many different types. Four strategies were performed to decipher the possible effective genes. First, assuming that there was a modified gene in this family of patients, all of the heterozygous mutations on chromosome 13 shared by the three patients in Family 1006983, with a frequency of less than 0.0001 and functional mutations were selected (non-coding mutations may also be responsible for the possibility of modification, but due to the huge amount of data, so rare functional mutations were focused on first). Candidate mutation information was shown in Supplementary Table 3. Second, genes interacting with *GJB2* were analysed emphatically. Third, copy number variants (CNVs) of genes interacting with *GJB2* gene were analysed^1^ by selecting the 20M gene sequence before and after the target genes, with no related CNV were deciphered. Last, related genes located in the *GJB2* signal pathway were determined (Supplementary Table 4), and *TJP1* and *TUBA3E* were found to be most likely to be interacting with *GJB2*.

### RT-qPCR of GJB6

**FIGURE 4:**
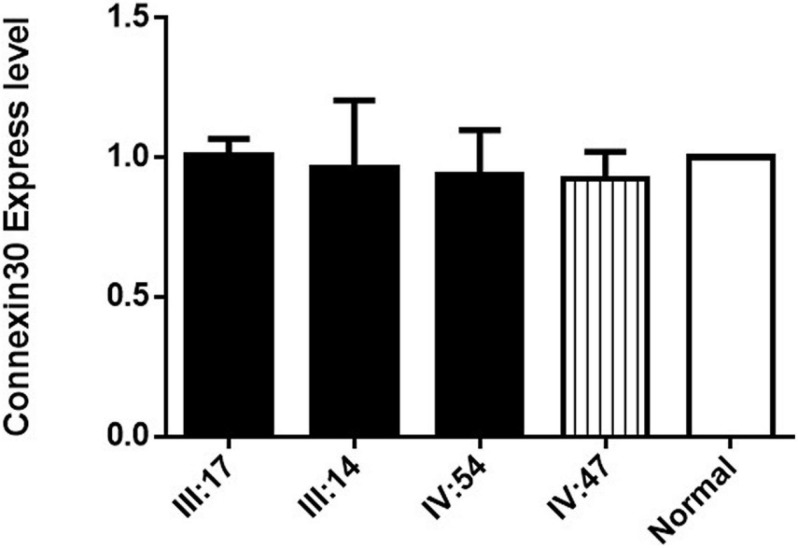
Connexin30 expression of some of the affected members and within-family normal controls from Family 1006983. III:17, III:14, and IV:54 are three hearing loss cases with the *GJB2* 235delC homozygous mutation, IV:47 is a normal control from Family 1006983. Normal represents the Connexin 30 expression of a person who is not a member of the family.

The expression of Connexin30 in the affected members of Family 1006983 showed no significant difference from that in the control group (Figure 4). The expression levels in the three sporadic cases revealed no consistent results, with only case1 having double expression compared to normal control (Figure 3C). Case 2 and case 3 showed no significant increase in the expression level from that of the normal controls or the heterozygous controls (Figures 3F,I).

## Discussion

### Phenotype-Genotype Correlation Analysis

There is striking phenotypic heterogeneity in the onset age as well as degree of hearing loss caused by *GJB2* mutations. For most genotypes, however, there is considerable variation. In clinic, cochlea developmental disorders are the mechanism of congenital hearing loss caused by Connexin26 deficiency, whereas the reduction of cochlea active amplification is the cause of late-onset hearing loss induced by Connexin26 deficiency (Mei et al., 2017). A multicenter genotype-phenotype correlation study showed that inactivating mutations cause a more severe phenotype than non-inactivating mutations (Snoeckx et al., 2005). Among c.235delC homozygotes, the majority do have pre-lingual profound hearing loss, while some c.235delC homozygotes have only post-lingual and/or moderate hearing loss. The phenotypic variances found among the cases recruited in this study are as follows.

#### Late-Onset Phenotype of Homozygous *GJB2* c.235delC

All of the patients from Family 1006983 and the three sporadic cases had late-onset hearing impairment. Previous studies of 295 control cases with homozygous *GJB2* c.235delC by our group reported that post-lingual cases accounted for 1.36% of the cohort, indicating a relatively rare variant (Zhao et al., 2011). Though relatively rare, the *GJB2* c.35delG homozygous mutation, which is the most prevalent mutation in Caucasians, could also contribute to late postnatal onset hearing loss (Pagarkar et al., 2006). There are also some *GJB2* pathogenic mutations associated with post-lingual hearing impairment, such as p.T55N, p.R75Q, p.D179N, and p.C202F (Morle et al., 2000; Primignani et al., 2003; Melchionda et al., 2005; Iossa et al., 2010), which are inherited as autosomal dominant patterns (Wang et al., 2017).

#### Milder Hearing Impairment Phenotype of Homozygous *GJB2* c.235delC

One affected member from Family 1006983 and three sporadic cases had moderate hearing loss, accounting for 50% of the eight cases recruited in this study, and the other four cases from Family 1006983 had severe to profound hearing loss. In fact, among the four severe to profound cases in Family 1006983, the testing age was 33, 59, 61, and 65 years, showing a progressive hearing impairment tendency according to the complaints of these patients.

#### *GJB2* Mutation and Sudden Hearing Loss

Case 2 complained of sudden hearing loss of the left ear when he came to our clinic on 14th September, 2015. In recent years, some studies have proposed that genetic deafness mutations may be associated with the pathogenesis of sudden hearing loss. Bora et al. once screened the *GJB2*, *GJB3*, and *GJB6* genes in 40 sudden hearing loss patients and 40 normal controls, but the connection between Connexin and sudden hearing loss could not be verified (Bora et al., 2010). In the Chinese population, previous studies showed that the incidence of the *GJB2* c.235delC mutation was low in sudden hearing loss patients (2.1%, 5/234) and had no significant difference from that of normal controls, suggesting that *GJB2* c.235delC has no correlation with sudden hearing loss (Zhan et al., 2014). In our group, we once screened the *GJB2* gene in 93 sudden hearing loss patients and 117 controls with normal hearing. Only two heterozygous c.235delC mutations were identified in the sudden hearing loss group, while five heterozygous c.235delC mutations were identified in the normal control group (data not published).

#### Genetic Counselling and Clinical Management

Genetic diagnosis and counselling is increasingly affecting the clinical practice of genetic hearing loss, especially genotype-phenotype correlation analysis. *GJB2* c.235delC is the most common deafness-related pathogenic variant in the Chinese population, and reporting the phenotypic heterogeneity of *GJB2* c.235delC homozygous cases is helpful throughout the genetic counselling procedure, and would contribute greatly to clinical practice. Except for providing information about incidence, recurrent risk and prevention strategies, phenotype heterogeneity should be accounted both before and after gene testing. For example, information if provided on the possible gene testing results and relevant phenotype prognosis before gene testing, phenotype heterogeneity should be taken into consideration; for the management of an individual with *GJB2* c.235delC homozygous mutation after gene testing, especially for a baby who has passed newborn hearing screening, audiology follow-up seems to be much more critical than performing cochlear implantation directly. Gene therapy as a potential clinical treatment method have been reported to be efficient in inherit hearing loss in animal models (Tan et al., 2019; Delmaghani and El-Amraoui, 2020; Niggemann et al., 2020). However, there are numerous challenges associated with *in vivo* gene therapy targeting the human inner ears to be addressed.

### Phenotypic Heterogeneity Mechanisms

Cellular and deafness mechanisms underlying *GJB2* induced hearing impairment are currently unclear, although numerous clinical reports indicate that Connexin26 mutations are associated with hearing impairment. Few studies have been performed on the pathogenesis of Connexin26 mutations. For example, in one clear functional study on a patient who had the Connexin26 c.35delG mutation and a p.E101G missense mutation, microscopic observation revealed nearly complete degeneration of hair cells and agenesis of the SV but no neural degeneration (Jun et al., 2000). In summary, previous studies on Connexin function showed that the cochlear development disorders lead to congenital deafness instead of hair cell degeneration and endocochlear potential reduction, while the reduction of active cochlear amplification leads to late-onset hearing loss, even though cochlear hair cells have no Connexin expression (Wingard and Zhao, 2015).

Most likely, the phenotypic heterogeneity is caused by modifier genes, but none has been identified to date. Modifier genes can be relatively easily identified in mice by crossing parental inbred strains that carry the disease-causing mutation and exhibit a difference in phenotype, such as the modifier gene *Moth1* in the Tubby mouse (Ikeda et al., 2002). However, it is difficult to perform linkage or association analysis to determine the modifier gene in human cases with only one family and a limited number of mild/moderate sporadic patients, such as the situation in this study. In our study, the targeted multi-gene sequence of one of the affected members identified no other variation except for homozygous *GJB2* c.235delC. Their identification could substantially contribute to a more accurate diagnosis and more appropriate genetic counselling (Hilgert et al., 2009b). Exome sequencing or genome sequencing may be better choice to find modifier genes in these unconditional cases. However, although we attempted different strategies, it is difficult to analyse the large amount of data of the genome sequencing, with numerous candidate genes being identified, but no further evidence was obtained to confirm the phenotypic correlation. Another family and many more sporadic cases with the *GJB2* c.235delC homozygous mutation and late-onset milder hearing impairment may provide the possibility to decipher the molecular mechanism. A WGA study may also an effective way to decipher the major modifier genes of *GJB2* c.235delC, as one had been previously performed on a separate set of *GJB2* c.35delG cases (Hilgert et al., 2009a). The WGA study needs a second set of cases to replicate the possible SNPs, however, *GJB2* c.235delC homozygote cases are limited.

From another point of view, we hypothesised that for some of the patients with more minor phenotypes than those with homozygous *GJB2* c.235delC, the overexpression of Connexin30 plays a compensatory role. The mutual pathogenic mechanism of the biallelic *GJB2* and *GJB6* genes is unclear. These two genes have 77% sequence homology. Connexin26 and Connexin30, which are encoded by these two genes, assemble into a complete heterologous gap junction channel together (Liu et al., 2009), playing a key role in inner ear K^+^ regulation. *GJB2* overexpression can effectively restore hearing impairment in *GJB6* knockout mice (Ahmad et al., 2007), suggesting that genetic correction by overexpression may restore hearing function effectively. We found some of the cases overexpressed Connexin3, however, we could not find consistency between the family and sporadic patients in this study. In addition, the total RNA extracted from whole blood could not verify the located expression of Connexin30. Furthermore, biopsy of the human cochlear is impossible for now, and measurement of the gene transcription of Connexins by RT-qPCR and of the protein expression of Connexins by western blots analysis cannot be achieved. We will continue focus on the subjects with the same phenotype in this study, to find out the underlying mechanism of the phenotype heterogeneity. In addition, animal experiments are still needed to find out whether increasing the Connexin30 protein level in Gjb2 knock out background could restore hearing. To all knowledge, there is no data to confirm that up-regulation of Connexin6 expression in Gjb2 knockout mice could not rescue the hearing up to date.

## Conclusion

In conclusion, we reported a family and three sporadic patients with homozygous c.235delC, who had an unconditional phenotype with post-lingual and/or moderate hearing impairment. Multi-genes sequencing showed no other pathogenic mutations or modifier genes. RT-qPCR of Connexin30 expression revealed that some of these cases overexpressed Connexin30, which may play a compensatory role in hearing impairment. However, the mechanism of the phenotypic heterogeneity and the specific mutation-induced pathological changes *in vivo* remain unclear, and there is little information is available for humans. Many more experiments and large series are needed to decipher the genetic code to extend this study in the future.

## Data Availability Statement

The original contributions presented in the study are included in the article/Supplementary Material, further inquiries can be directed to the corresponding authors.

## Ethics Statement

The studies involving human participants were reviewed and approved by the study was approved by the Committee of Medical Ethics of the Chinese PLA General Hospital. The patients/participants provided their written informed consent to participate in this study. Written informed consent was obtained from the individual(s) for the publication of any potentially identifiable images or data included in this article.

## Author Contributions

HW and YG conceived and designed the experiments. HW, YG, JG, LL, and JY performed the experiments. HW, JG, WX, LX, and LY analysed the data. JY and CZ contributed reagents, materials, and analysis tools. HW wrote the manuscript. DW and QW performed the critical reading and discussion of manuscript. All authors contributed to the article and approved the submitted version.

## Conflict of Interest

The authors declare that the research was conducted in the absence of any commercial or financial relationships that could be construed as a potential conflict of interest.

## Footnotes

1 https://glm.genecards.org/#results

## Supplementary Material

Supplementary Figure 1 Flow chart to state the outline of the manuscript.

Supplementary Figure 2 Quantitative condition of RT-qPCR.

Supplementary Table 1 The total amount of the whole genome sequencing data of Family 1006983.

Supplementary Table 2 The whole genome sequencing depth and coverage of Family 1006983.

Supplementary Table 3 The candidate gene variants of whole genome sequencing from Family 1006983.

Supplementary Table 4 related genes located in the *GJB2* signal pathway (Whole genome sequencing data of Family 1006983).
