# Supplementary figures and images for: Phenotypic Heterogeneity of Post-lingual and/or Milder Hearing Loss for the Patients With the GJB2 c.235delC Homozygous Mutation
Source: Front Cell Dev Biol. 2021 Feb 26;9:647240. doi: 10.3389/fcell.2021.647240 (PMC7953049; doi:10.3389/fcell.2021.647240)

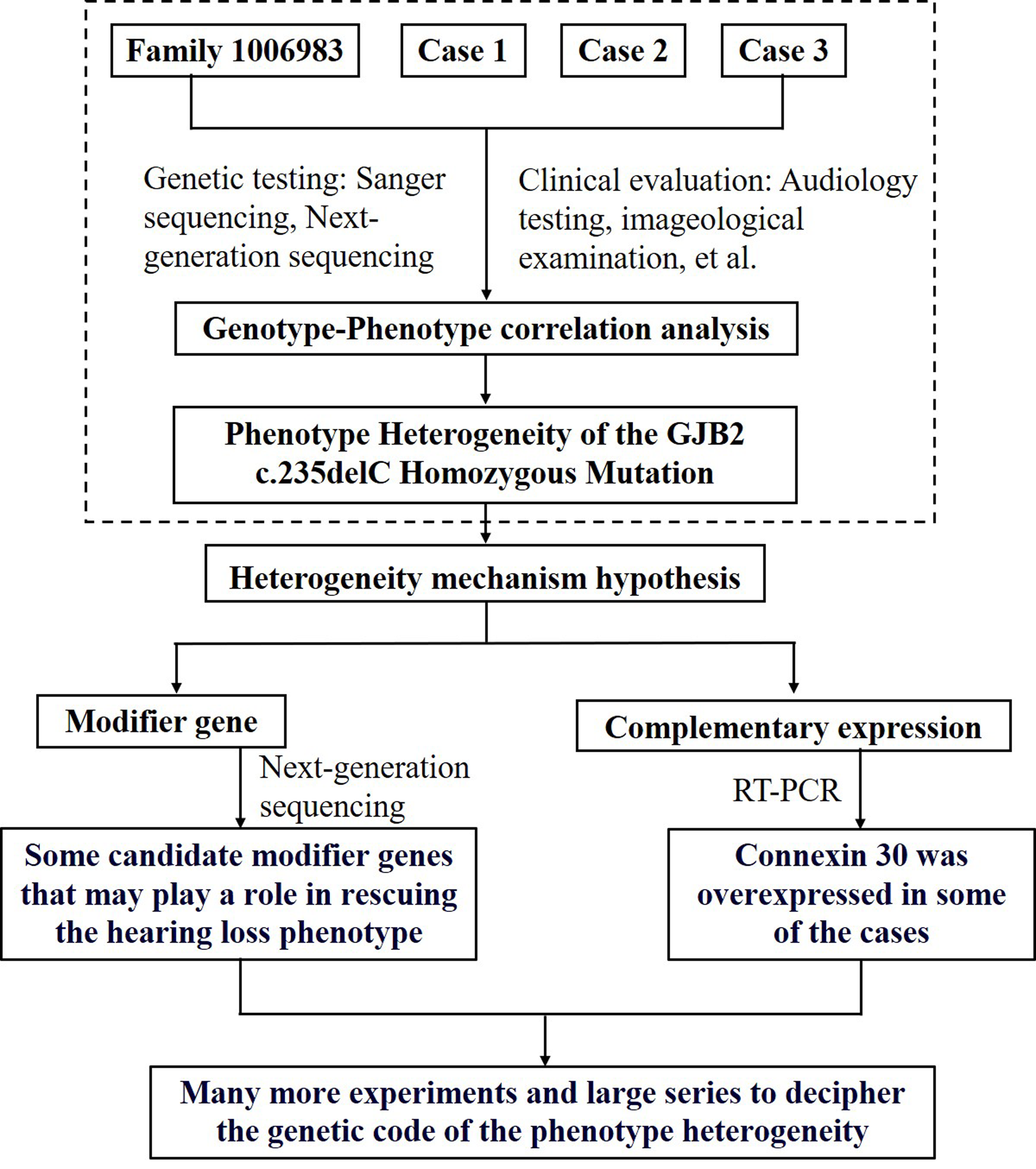

Supplement: Supplementary Figure 1 — Flow chart to state the outline of the manuscript. [file Image_1.TIF]

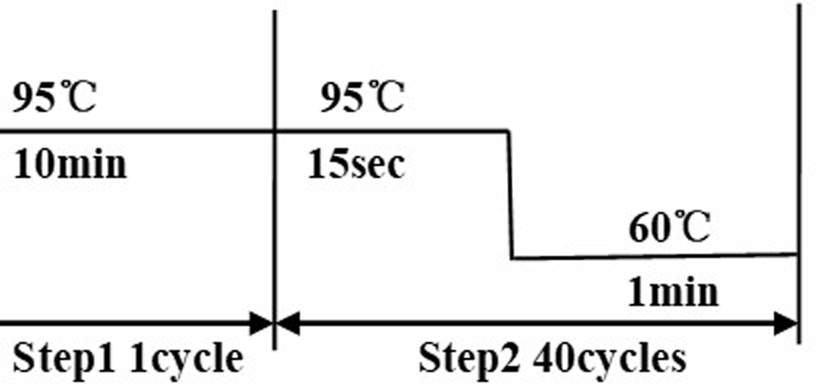

Supplement: Supplementary Figure 2 — Quantitative condition of RT-qPCR. [file Image_2.TIF]
