# Supplementary material for: Phenotypic Heterogeneity of Post-lingual and/or Milder Hearing Loss for the Patients With the GJB2 c.235delC Homozygous Mutation
Source: Front Cell Dev Biol. 2021 Feb 26;9:647240. doi: 10.3389/fcell.2021.647240 (PMC7953049; doi:10.3389/fcell.2021.647240)
Supplement: Supplementary Table 1 — The total amount of the whole genome sequencing data of Family 1006983. [file Data_Sheet_1.docx]

Table S1. The total amount of the whole genome sequencing data of Family 1006983

| Data type | Amount |
| --- | --- |
| Lines of input read | 7132735 |
| Variants processed | 7132601 |
| Variants remaining after filtering | 7132601 |
| Lines of output written | 7132601 |
| Novel / existing variants | 472675 (6.6%) / 6659926 (93.4%) |
| Overlapped genes | 85297 |
| Overlapped transcripts | 273078 |
| Overlapped regulatory features | 169588 |

Note: Lines of input read” refers to the number of reads input VEP for analysis (http://asia.ensembl.org/info/docs/tools/vep/vep_formats.html); The overlapped genes here Includes non-coding genes and pseudogenes.

Table S2. The whole genome sequencing depth and coverage of Family 1006983

|  | III:13 | III:17 | III:40 | III:14 | IV:44 | IV:47 |
| --- | --- | --- | --- | --- | --- | --- |
| Average sequencing depth (X) | 44.77 | 43.22 | 44.13 | 40.74 | 44.28 | 45.12 |
| Coverage (%) | 91.97 | 91.83 | 92.37 | 92.34 | 92.36 | 92.36 |
| Coverage at least 4X (%) | 91.67 | 91.7 | 92.27 | 92.18 | 92.27 | 92.28 |
| Coverage at least 10X (%) | 91.36 | 91.44 | 91.93 | 91.18 | 91.91 | 91.98 |
| Coverage at least 20X (%) | 90.49 | 90.69 | 89.68 | 85.23 | 89.31 | 89.63 |
| Coverage at least 30X (%) | 87.51 | 86.11 | 82.62 | 72.75 | 81.66 | 83.13 |
| Coverage at least 40X (%) | 75.05 | 65.08 | 68.27 | 53.17 | 64.49 | 67.74 |

Table S3. The candidate gene variants of whole genome sequencing from Family 1006983

| Chr | position | ID | Variant type^1^ | Consequence | Gene | Function |
| --- | --- | --- | --- | --- | --- | --- |
| 13 | 21563345 | . | snv | Misse nse_variant | *LATS2* | protein_coding |
| 13 | 23910868 | . | snv | missense_variant | *SACS* | protein_coding |
| 13 | 25073449 | . | snv | missense_variant | *PARP4* | protein_coding |
| 13 | 25116246 | . | snv | missense_variant | *LOC101927375* | protein_coding |
| 13 | 28001284 | rs192739102 | snv | missense_variant | *GTF3A* | protein_coding |
| 13 | 33851572 | rs201578696 | indel | splice_region_variant&intron_variant&non_coding_transcript_variant | *STARD13-AS* | misc_RNA |
| 13 | 33851574 | rs200279333 | indel | splice_region_variant&intron_variant&non_coding_transcript_variant | *STARD13-AS* | misc_RNA |
| 13 | 46850757 | . | indel | frameshift_variant | *LRRC63* | protein_coding |
| 13 | 49746186 | rs199523846 | snv | missense_variant | *FNDC3A* | protein_coding |
| 13 | 101944567 | rs374004710 | snv | splice_region_variant&intron_variant | *NALCN* | protein_coding |
| 13 | 111995219 | rs147238221 | snv | missense_variant | *TEX29* | protein_coding |

1. snv, single nuclear variant; indel, insert or deletion

| **Query gene id** | **Query gene** | **hit location** | **Partner candidate id** | **Total Score** | **SuperPathway** | **Weight=1** | **Conclusion** |
| --- | --- | --- | --- | --- | --- | --- | --- |
| 69191 | *GJB2* | 15 | *TJP1* | 0.4940092 | 0.4940092 | 1.447907 | 1 inframe shift |
| 69191 | *GJB2* | 99 | *TUBA3E* | 0.2457297 | 0.2457297 | 1.447907 | 1 splice-site variant, shared by cases |
| 69191 | *GJB2* | 8 | *GJC1* | 0.6145398 | 0.6145398 | 1.447907 | 1 non-coding variant, shared by cases |
| 69191 | *GJB2* | 22 | *PRKCA* | 0.3662603 | 0.3662603 | 1.447907 | 1 non-coding variant, shared by cases |
| 69191 | *GJB2* | 31 | *CLTB* | 0.2482795 | 0.2482795 | 1.447907 | 1 non-coding variant, shared by cases and controls |
| 69191 | *GJB2* | 49 | *ADCY9* | 0.2457978 | 0.2457978 | 1.447907 | 2 non-coding variant, shared by cases and controls |
| 69191 | *GJB2* | 55 | *GNAQ* | 0.2457978 | 0.2457978 | 1.447907 | 2 non-coding variant, shared by cases and controls |
| 69191 | *GJB2* | 72 | *GUCY1A3* | 0.2457978 | 0.2457978 | 1.447907 | 1 non-coding variant, shared by cases |
| 69191 | *GJB2* | 87 | *SP1* | 0.2457978 | 0.2457978 | 1.447907 | 1 non-coding variant, shared by cases |
| 69191 | *GJB2* | 89 | *BRAF* | 0.2457297 | 0.2457297 | 1.447907 | 1 non-coding variant, shared by cases |
| 69191 | *GJB2* | 94 | *TUBA1A* | 0.2457297 | 0.2457297 | 1.447907 | 2 non-coding variant, shared by cases |

Table S4. related genes located in the *GJB2* signal pathway (Whole genome sequencing data of Family 1006983)
